# Prognostic nutritional index predicts staging and mortality in cardiovascular-kidney-metabolic syndrome

**DOI:** 10.1371/journal.pone.0338084

**Published:** 2025-12-18

**Authors:** Yiying Liu, Fuyuan Zhang, Ruikang Liu, Jun Li, Cong Chen, Xuanchun Huang, Yulian Yuan, Chao Meng, Xiao Xia, Yonghao Li

**Affiliations:** 1 Department of Cardiology, Guang’anmen Hospital, China Academy of Chinese Medical Sciences, Beijing, China; 2 School of Medicine, Tsinghua University, Beijing, China; Shuguang Hospital, CHINA

## Abstract

**Background:**

Cardiovascular-kidney-metabolic syndrome (CKM) is a multisystem disorder involving cardiovascular, kidney, and metabolic diseases, associated with high mortality risk. The prognostic nutritional index (PNI), reflecting nutritional and immune status, predicts outcomes in various diseases. This study evaluates PNI’s association with CKM stages and mortality.

**Methods:**

Data from 10,448 CKM patients in the 1999–2018 NHANES cohort were analyzed. Weighted logistic regression assessed PNI’s relation to CKM stages. Nonlinear relationships were explored via restricted cubic spline and two-piecewise linear regression. Kaplan-Meier and Cox models examined PNI’s association with all-cause and cardiovascular mortality. Subgroup analyses explored interactions with demographic and clinical factors. Significance was set at *P* < 0.05.

**Results:**

PNI was highest in CKM stage 0 and lowest in stage 4 (53.52 ± 0.16 vs. 51.54 ± 0.25, *P* < 0.001). Notably, an inverted U-shaped relationship was identified for early stages (0–1), while an inverse association was observed for stage 4 and advanced CKM. Over a median 123-month follow-up, the highest PNI quartile had superior survival for all-cause (83.63% vs. 52.58%, *P* < 0.001) and cardiovascular mortality (95.69% vs. 88.18%, *P* < 0.001). PNI was inversely correlated with mortality risk, with specific thresholds observed at 50 for all-cause mortality and 51 for cardiovascular mortality. This association was modified by gender, race, and BMI.

**Conclusion:**

PNI is significantly associated with both early and advanced stages of CKM. PNI serves as an independent predictor for all-cause and cardiovascular mortality in CKM patients.

## 1 Introduction

Cardiovascular-kidney-metabolic (CKM) syndrome is a novel concept introduced by the American Heart Association (AHA), highlighting the interconnectedness of cardiovascular, renal, and metabolic diseases, which together contribute to multi-organ dysfunction and an increased incidence of cardiovascular events [1]. In recent years, the prevalence of CKM syndrome has risen among American adults, particularly in populations with lower socioeconomic status [2]. Furthermore, the severity of CKM is strongly associated with all-cause mortality, with women exhibiting a notably higher risk of death [3]. The high prevalence of CKM is closely tied to various pathophysiological mechanisms, including insulin resistance, hypertension, and dyslipidemia, which collectively elevate the risk of cardiovascular events and kidney disease [4]. Due to the multisystem nature of CKM, its diagnosis requires a comprehensive assessment of cardiovascular, renal, and metabolic indicators [5]. Traditional biomarkers lack the specificity and sensitivity necessary for accurate CKM diagnosis, as they fail to capture pathological changes across multiple systems. As a result, researchers are investigating novel combinations of biomarkers to improve diagnostic accuracy and facilitate early detection [6]. To better predict mortality risk in CKM patients, several new predictive models have been developed [7,8]. Additionally, emerging evidence suggests that novel biomarkers not only help identify high-risk CKM individuals but also support early interventions, ultimately improving long-term health outcomes for CKM patients [9–13].

In recent years, the prognostic nutritional index (PNI), a comprehensive indicator reflecting both nutritional status and immune function, has proven to be an effective prognostic predictor in various diseases [14]. In heart failure patients, PNI has been independently and significantly associated with both all-cause and cardiovascular mortality, demonstrating its potential in predicting cardiovascular events [15]. A separate study has further confirmed that PNI can serve as a key indicator for assessing cardiovascular disease risk [16]. Additionally, PNI is strongly associated with disease progression in chronic kidney disease (CKD) patients, particularly those with concurrent chronic heart failure, where PNI functions as a clinical biomarker for disease progression [17]. PNI also proves effective in predicting nutritional status and disease risk in metabolic disorders. It is linked to the onset and progression of metabolic syndrome, diabetes, and other conditions, with lower PNI values potentially indicating a higher risk for these diseases [18]. However, to date, no population-based study has specifically explored the associations between PNI and different stages of CKM syndrome. Likewise, there has been no population-based study systematically evaluating the predictive value of PNI for all-cause and cardiovascular mortality in CKM populations.

The National Health and Nutrition Examination Survey (NHANES) is a major national survey in the United States designed to collect data on the health and nutritional status of the U.S. population. NHANES collects data through interviews, standardized physical exams, and biological sample collection. The strength of this data lies in its comprehensiveness and representativeness, covering a wide range of health indicators and nutritional statuses, with a nationally representative sample [19]. The rich data from NHANES provides a strong foundation for research on PNI and CKM, enabling the exploration of the complex relationship between nutritional status and cardiovascular health.

## 2 Methods

### 2.1 Study design and participants

NHANES employs a biennial cross-sectional design and a complex multistage probability sampling method to collect extensive data on the nutritional and health status of the U.S. population. For this analysis, publicly available NHANES datasets from 1999 to 2018 were used, with data collected in accordance with ethical guidelines and informed consent obtained from all participants. Comprehensive details on the study design and data collection methods can be found on the official website (www.cdc.gov/nchs/nhanes/). All research procedures followed ethical standards and relevant regulations.

**Fig 1:**
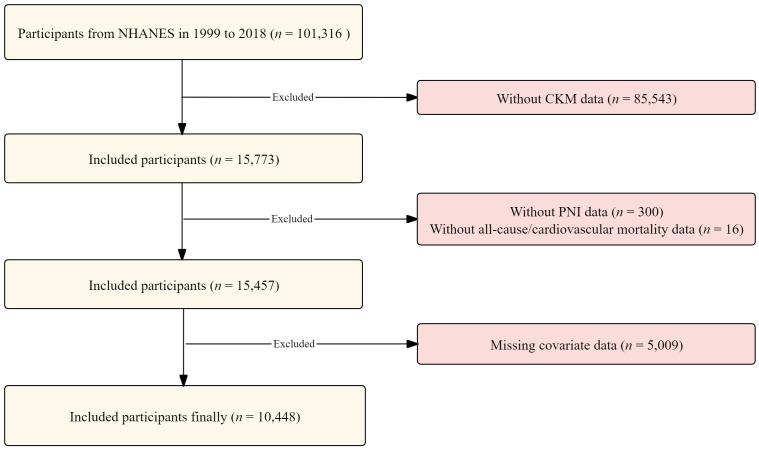
Study selection flow chart.

For this study, the NHANES dataset from 1999 to 2018 was analyzed using a cross-sectional cohort design. Initially, 101,316 participants were included. The following exclusions were applied: 85,543 participants under the age of 18 or lacking data on CKM syndrome, 316 participants with incomplete PNI or mortality data, and 5,009 participants with missing data on key covariates. After applying these criteria, 10,448 eligible participants remained for the final analysis. A detailed overview of the participant screening process is presented in Fig 1.

### 2.2 Stages of CKM syndrome

According to the latest definition and staging of CKM provided by AHA, CKM syndrome is classified into the following stages [20,21]:

CKM stage 0 represents the absence of CKM-related health risk factors (e.g., overweight/obesity, hypertension, hypertriglyceridemia, metabolic syndrome, diabetes mellitus, CKD, or cardiovascular disease [CVD]). CKM stage 1 is characterized by excessive or dysfunctional obesity without other metabolic risk factors or CKD (body mass index [BMI] ≥ 25 kg/m², including BMI ≥ 23 kg/m² for Asians; waist circumference ≥ 88 cm for women and ≥ 102 cm for men, with Asian women having a waist circumference ≥ 80 cm and Asian men ≥ 90 cm; fasting blood glucose of 100–124 mg/dL or HbA1c between 5.7% and 6.4%). CKM stage 2 involves the presence of metabolic risk factors (e.g., hypertriglyceridemia, hypertension, metabolic syndrome, diabetes mellitus) or CKD. CKM stage 3 indicates a very high risk of chronic kidney disease (G4 or G5 CKD, or very high risk according to the KDIGO classification) or a high predicted 10-year risk of cardiovascular disease. CKM stage 4 is characterized by the presence of clinical cardiovascular disease (e.g., coronary heart disease, heart failure, stroke, peripheral arterial disease, atrial fibrillation) in individuals with excessive or dysfunctional obesity, other metabolic risk factors, or CKD. Stages 3 and 4 are collectively referred to as advanced CKM, encompassing patients diagnosed with or at high risk for cardiovascular disease.

The CKM syndrome staging framework was applied retrospectively to the NHANES 1999–2018 cohorts. This is methodologically sound because the diagnostic criteria for all components required for staging were stable and consistently available in the NHANES data throughout the study period, allowing for accurate assignment to CKM stages as defined by the 2023 AHA criteria. Detailed staging criteria are provided in S1 Table.

### 2.3 Calculation of PNI

The formula for calculating PNI is given by: PNI= (10 × serum albumin) + (0.005 × lymphocyte count) [22]. Serum albumin was measured in grams per deciliter (g/dL), and lymphocyte count was measured in cells per microliter (µL). This formula was chosen due to its simplicity, clinical applicability, and compatibility with major public health databases like NHANES. It directly uses the original units for serum albumin and lymphocyte count without requiring conversions, making it easy to apply in clinical settings and ensuring reproducibility across studies. Detailed information on the laboratory procedures can be found on the NHANES official website. Participants were categorized into four groups based on PNI quartiles: Q1 (30–50], Q2 (50–52.5], Q3 (52.5–55.5], and Q4 (55.5–262.5], with Q1 serving as the reference group.

### 2.4 Assessment methods of mortality

The primary outcomes of this study were all-cause mortality and cardiovascular mortality. Mortality data were obtained from the National Death Index (NDI), which includes death certificate records from the National Center for Health Statistics (NCHS), with the most recent update on December 31, 2018. Causes of death were classified according to ICD-10 codes. All-cause mortality encompassed deaths from any cause, including heart disease (codes 054–068), malignant tumors (codes 019–043), accidents (codes 112–123), cerebrovascular disease (code 070), diabetes (code 046), and other causes. Cardiovascular mortality, throughout the follow-up period, referred to deaths caused by heart disease. The follow-up period extended from the initial visit to either the date of death or December 31, 2018.

### 2.5 Covariates

This study analyzed demographic and behavioral factors using data from the NHANES questionnaire and physical examination. Demographic factors included age, gender, race, poverty-to-income ratio (PIR), education, and marital status, while behavioral factors encompassed smoking, alcohol consumption, and physical activity. Smoking status was assessed using self-reported data and serum cotinine levels. Nonsmokers were defined as individuals who reported being ‘never smokers’ and had serum cotinine levels below 0.05 ng/ml, while smokers were those who reported being ‘current smokers’ or had serum cotinine levels of 0.05 ng/ml or higher [23]. Drinking status was defined as consuming ≥ 4 alcoholic beverages per day (including occasional heavy drinkers and chronic drinkers) versus consuming < 4 beverages per day, with the latter group classified as non-drinkers [24]. Physical activity levels were assessed using a questionnaire on activities from the past 30 days, with each activity assigned a MET value based on its type and intensity. Physical activity was categorized as low for activities under 500 MET minutes per week and high for those equal to or exceeding 500 MET minutes per week [25].

Body mass index (BMI) was calculated from NHANES physical examination data using the formula BMI = W/H², where W represents weight in kilograms and H represents height in meters [26]. Hypertension, hyperlipidemia, and diabetes were identified based on self-reported medical histories. Hypertension was defined as a history of high blood pressure or self-reported systolic blood pressure (SBP) exceeding 140 mmHg or diastolic blood pressure (DBP) exceeding 90 mmHg. Hyperlipidemia was defined as total cholesterol (TC) ≥ 200 mg/dL, triglycerides (TG) ≥ 150 mg/dL, low-density lipoprotein cholesterol (LDL-C) ≥ 130 mg/dL, or high-density lipoprotein cholesterol (HDL-C) ≤ 40 mg/dL for men or ≤ 50 mg/dL for women. Additionally, participants taking lipid-lowering medications were also considered to have hyperlipidemia [27]. Diabetes was identified based on a history of the condition, use of insulin or diabetic medications, hemoglobin A1c levels ≥ 6.5%, fasting blood glucose levels ≥ 126 mg/dL, or postprandial blood glucose levels ≥ 200 mg/dL two hours after eating.

### 2.6 Statistical analysis

All models were adjusted for relevant covariates, and data analysis was conducted using R software (version 4.4.1). Weighted statistical analyses were performed for all participants, accounting for the complex multistage stratified sampling design of NHANES [28]. Between-group differences in categorical variables were assessed using weighted chi-square tests, while differences in continuous variables were analyzed with weighted t-tests and analysis of variance (ANOVA).

To examine the association between PNI and CKM syndrome, both univariate and multivariate weighted logistic regression models were applied. Three specific models were constructed. Nonlinear relationships between PNI and CKM were assessed using smoothed curve fitting, and potential threshold effects were explored through a two-stage linear regression model. Kaplan-Meier analysis was performed to investigate the association between PNI and all-cause, cardiovascular, and diabetes-related mortality in advanced CKM patients. A substantial intersection of the curves with a *P*-value greater than 0.05 suggests temporal variation in the hazard ratio, indicating a violation of the proportional hazards assumption. If this assumption held, subsequent Cox regression analysis was performed. Weighted logistic regression models, both univariate and multivariate, were used to investigate the relationship between PNI and CKM syndrome. Nonlinear relationships were assessed using multivariable-adjusted restricted cubic spline (RCS) models with three knots. Subgroup analyses, stratified by gender, race, age, and other key variables, were conducted to examine interactions between PNI and these variables concerning various mortality outcomes in CKM. Statistical significance was defined as *P* < 0.05.

### 2.7 Ethical approval and consent to participate

This study utilized the publicly available NHANES dataset, ensuring ethical compliance and obtaining explicit consent from participants. The research adhered to all relevant standards and regulations throughout the process.

## 3 Results

### 3.1 Baseline characteristics of study participants

#### 3.1.1 Baseline characteristics of study participants with CKM syndrome staging.

Table 1 presents the baseline characteristics of participants categorized by CKM syndrome stages. The cohort included 10,448 individuals: 871 (8.3%) in Stage 0, 1,446 (13.8%) in Stage 1, 6,355 (60.8%) in Stage 2, 659 (6.3%) in Stage 3, and 1,117 (10.7%) in Stage 4. BMI was highest in Stage 3 (30.73 ± 0.24) and lowest in Stage 0 (21.52 ± 0.09), with a significant trend across stages (*P* < 0.001). The percentage of individuals with BMI > 30 was highest in Stage 3 (53.79%), while Stage 0 had the highest proportion of those with BMI < 25 (95.07%). Mean age increased with CKM stage, from 33.58 years in Stage 0 to 63.89 years in Stage 4 (*P* < 0.001). PIR was highest in Stage 3 (3.35 ± 0.08) and lowest in Stage 4 (2.78 ± 0.07, *P* < 0.001). PNI declined with advancing stages, with Stage 0 having the highest PNI (53.52 ± 0.16) and Stage 4 the lowest (51.54 ± 0.25, *P* < 0.001). Stage 4 had the highest proportion of males (57.03%) compared to Stage 0 (33.12%, *P* < 0.001). The majority of participants were Non-Hispanic White, particularly in Stage 4 (78.07%), while Non-Hispanic Black participants were more common in Stage 0 (12.77%). Marital status, smoking, and physical activity varied significantly across stages (*P* < 0.001). The prevalence of hypertension and diabetes increased with CKM stage, with Stage 4 showing the highest rates in each category (*P* < 0.001). Hyperlipidemia was most prevalent in Stage 4 (90.12%, *P* < 0.001). All-cause mortality was lowest in Stage 0 (1.25%) and highest in Stage 4 (38.08%, *P* < 0.001), with a similar trend observed for cardiovascular mortality.

**Table 1:** Baseline characteristics of NHANES 1999-2018 participants grouped by CKM syndrome.

Variable	Total (*n* = 10,448)	CKM Stage 0 (*n* = 871)	CKM Stage 1 (*n* = 1,446)	CKM Stage 2 (*n* = 6,355)	CKM Stage 3 (*n* = 659)	CKM Stage 4 (*n* = 1,117)	*P*
**Age, mean ± se**	47.51 ± 0.29	33.58 ± 0.47	41.55 ± 0.57	48.33 ± 0.30	52.61 ± 0.57	63.89 ± 0.55	<0.001
**PIR, mean ± se**	3.08 ± 0.04	3.16 ± 0.08	3.11 ± 0.07	3.07 ± 0.04	3.35 ± 0.08	2.78 ± 0.07	<0.001
**PNI, mean ± se**	52.65 ± 0.07	53.52 ± 0.16	52.93 ± 0.11	52.60 ± 0.09	52.61 ± 0.21	51.54 ± 0.25	<0.001
**Body mass index, n (%)**							<0.001
<25	3118 (31.59)	791 (95.07)	440 (29.82)	1555 (25.31)	75 (7.91)	257 (23.19)	
25-30	3514 (33.10)	47 (3.09)	637 (45.67)	2210 (34.41)	247 (38.30)	373 (32.82)	
>30	3816 (35.31)	33 (1.84)	369 (24.51)	2590 (40.28)	337 (53.79)	487 (43.99)	
**Age, n(%)**							<0.001
<40	3335 (35.30)	639 (71.65)	687 (48.63)	1874 (32.43)	94 (16.84)	41 (4.79)	
40-60	3475 (39.11)	191 (23.98)	536 (38.91)	2194 (41.55)	307 (56.18)	247 (27.59)	
>60	3638 (25.59)	41 (4.37)	223 (12.46)	2287 (26.02)	258 (26.98)	829 (67.62)	
**Poverty Income Ratio, n (%)**							<0.001
<1.5	3510 (23.47)	289 (22.23)	476 (22.50)	2106 (23.56)	204 (20.16)	435 (28.27)	
1.5-3	2642 (24.14)	191 (21.10)	341 (23.66)	1650 (24.44)	143 (20.15)	317 (29.14)	
>3	4296 (52.39)	391 (56.67)	629 (53.84)	2599 (52.00)	312 (59.69)	365 (42.59)	
**Prognostic nutritional index, n(%)**							<0.001
<50	2872 (24.97)	148 (14.68)	310 (20.70)	1778 (25.58)	187 (25.02)	449 (39.54)	
50-52.5	2370 (22.90)	215 (26.06)	340 (23.45)	1424 (22.67)	145 (21.69)	246 (20.87)	
52.5-55.5	2629 (26.27)	245 (28.88)	417 (29.24)	1561 (25.54)	169 (27.98)	237 (22.10)	
>55.5	2577 (25.86)	263 (30.39)	379 (26.62)	1592 (26.21)	158 (25.32)	185 (17.50)	
**Gender, n (%)**							<0.001
Male	5373 (51.54)	303 (33.12)	749 (53.64)	3120 (49.75)	549 (84.62)	652 (57.03)	
Female	5075 (48.46)	568 (66.88)	697 (46.36)	3235 (50.25)	110 (15.38)	465 (42.97)	
**Race, n (%)**							<0.001
Mexican American	1612 (7.15)	81 (4.63)	255 (10.10)	1088 (7.48)	82 (5.51)	106 (3.89)	
Other Hispanic	797 (4.46)	60 (4.41)	143 (6.03)	474 (4.39)	54 (3.50)	66 (3.00)	
Non-Hispanic White	5333 (72.54)	426 (70.78)	675 (69.42)	3194 (72.52)	351 (75.18)	687 (78.07)	
Non-Hispanic Black	1901 (10.13)	193 (12.77)	219 (8.76)	1148 (10.01)	127 (9.95)	214 (10.40)	
Other Race – Including Multi-Racial	805 (5.72)	111 (7.41)	154 (5.69)	451 (5.60)	45 (5.85)	44 (4.64)	
**Marital status, n (%)**							<0.001
Married	5743 (58.46)	340 (44.29)	784 (58.86)	3551 (58.93)	439 (69.30)	629 (62.63)	
Widowed	840 (5.52)	16 (1.11)	43 (2.15)	543 (5.97)	32 (2.93)	206 (14.94)	
Divorced	1082 (9.90)	53 (5.79)	131 (8.98)	685 (10.58)	67 (10.47)	146 (11.06)	
Separated	315 (2.09)	21 (1.83)	41 (2.05)	205 (2.27)	20 (1.63)	28 (1.61)	
Never married	1710 (16.73)	365 (36.87)	299 (19.12)	920 (15.27)	60 (10.45)	66 (4.58)	
Living with partner	758 (7.30)	76 (10.11)	148 (8.85)	451 (6.98)	41 (5.22)	42 (5.18)	
**Smoking status, n (%)**							<0.001
No	5573 (53.45)	584 (65.46)	850 (57.76)	3406 (53.62)	296 (45.26)	437 (37.40)	
Yes	4875 (46.55)	287 (34.54)	596 (42.24)	2949 (46.38)	363 (54.74)	680 (62.60)	
**Education, n (%)**							<0.001
Less than high school	2440 (15.85)	105 (8.83)	294 (13.81)	1532 (16.19)	154 (15.43)	355 (25.18)	
High school or equivalent	2373 (22.61)	162 (18.80)	285 (19.04)	1474 (23.01)	171 (28.41)	281 (26.03)	
College or above	5635 (61.54)	604 (72.37)	867 (67.16)	3349 (60.80)	334 (56.16)	481 (48.79)	
**Drinking status, n (%)**							0.002
No	2854 (22.75)	211 (20.37)	348 (20.04)	1806 (23.36)	147 (20.75)	342 (27.34)	
Yes	7594 (77.25)	660 (79.63)	1098 (79.96)	4549 (76.64)	512 (79.25)	775 (72.66)	
**Physical Activity Equivalent, n(%)**							<0.001
Low physical activity;	3916 (34.50)	229 (23.80)	420 (27.38)	2475 (36.73)	234 (30.76)	558 (46.08)	
High physical activity	6532 (65.50)	642 (76.20)	1026 (72.62)	3880 (63.27)	425 (69.24)	559 (53.92)	
**Hypertension, n (%)**							<0.001
No	5781 (59.55)	871 (100.00)	1446 (100.00)	3194 (53.75)	8 (0.84)	262 (27.48)	
Yes	4667 (40.45)	0 (0.00)	0 (0.00)	3161 (46.25)	651 (99.16)	855 (72.52)	
**Diabetes, n (%)**							<0.001
No	8391 (85.03)	866 (99.65)	1414 (98.20)	5056 (84.19)	414 (72.39)	641 (61.27)	
Yes	2057 (14.97)	5 (0.35)	32 (1.80)	1299 (15.81)	245 (27.61)	476 (38.73)	
**Hyperlipidemia, n(%)**							<0.001
No	2560 (25.29)	590 (67.51)	618 (41.99)	1181 (18.43)	55 (6.54)	116 (9.88)	
Yes	7888 (74.71)	281 (32.49)	828 (58.01)	5174 (81.57)	604 (93.46)	1001 (90.12)	
**All-cause mortality, n(%)**							<0.001
No	8846 (88.32)	857 (98.75)	1378 (96.53)	5458 (88.90)	520 (84.03)	633 (61.92)	
Yes	1602 (11.68)	14 (1.25)	68 (3.47)	897 (11.10)	139 (15.97)	484 (38.08)	
**Cardiovascular mortality, n(%)**							<0.001
No	9949 (96.53)	869 (99.88)	1433 (99.35)	6096 (97.04)	629 (95.93)	922 (85.00)	
Yes	499 (3.47)	2 (0.12)	13 (0.65)	259 (2.96)	30 (4.07)	195 (15.00)	

Weighted means were calculated for continuous variables, and categorical variables were expressed as n (%). The chi-square test or Fisher’s exact test was used for comparisons between groups, with *P* < 0.05 considered statistically significant. Q, quartile. CKM, Cardiovascular-kidney-metabolic syndrome. BMI, body mass index. PIR, poverty income ratio.

PNI, prognostic nutritional index.

#### 3.1.2 Baseline characteristics of study participants stratified by PNI quartiles.

Table 2 presents the baseline characteristics of participants stratified by PNI quartiles. The cohort included 10,448 participants: 2,872 (27.5%) in PNI Q1, 2,370 (22.7%) in PNI Q2, 2,629 (25.2%) in PNI Q3, and 2,577 (24.7%) in PNI Q4. BMI was highest in PNI Q1 (30.01 ± 0.17) and lowest in PNI Q4 (27.72 ± 0.19, *P* < 0.001). Age was highest in PNI Q1 (53.94 ± 0.43) and lowest in PNI Q4 (41.13 ± 0.47, *P* < 0.001). The poverty-to-income ratio (PIR) was highest in PNI Q3 (3.15 ± 0.05) and lowest in PNI Q4 (2.87 ± 0.05, *P* < 0.001). A higher proportion of males was observed in PNI Q4 (65.17%) compared to PNI Q1 (39.05%, *P* < 0.001). Non-Hispanic Whites represented the majority in all quartiles, with the highest proportion in PNI Q1 (74.65%) and the lowest in PNI Q4 (68.67%). Marital status, smoking, and drinking habits varied significantly across quartiles (*P* < 0.001). Hypertension was most prevalent in PNI Q4 (67.46%) and least in PNI Q1 (50.01%, *P* < 0.001), while diabetes was lowest in PNI Q4 (12.22%, *P* < 0.001). Hyperlipidemia was similarly distributed across quartiles (*P* = 0.818). All-cause mortality was highest in PNI Q1 (20.01%) and lowest in PNI Q4 (7.76%, *P* < 0.001). Cardiovascular mortality also decreased with higher PNI (*P* < 0.001).

**Table 2:** Baseline characteristics according to PNI quartiles.

Variable	Total (*n* = 10,448)	PNI Q1 (*n* = 2872)	PNI Q2 (*n* = 2370)	PNI Q3 (*n* = 2629)	PNI Q4 (*n* = 2,577)	*P*
**Age, mean ± se**	47.51** ± **0.29	53.94** ± **0.43	49.48** ± **0.44	45.95** ± **0.36	41.13** ± **0.47	<0.001
**PIR, mean ± se**	3.08** ± **0.04	3.08** ± **0.06	3.23** ± **0.05	3.15** ± **0.05	2.87** ± **0.05	<0.001
**CKM, n(%)**						<0.001
0	871 (9.89)	148 (5.81)	215 (11.25)	245 (10.87)	263 (11.62)	
1	1446 (14.93)	310 (12.38)	340 (15.29)	417 (16.62)	379 (15.38)	
2	6355 (59.99)	1778 (61.46)	1424 (59.39)	1561 (58.32)	1592 (60.81)	
3	659 (6.35)	187 (6.37)	145 (6.02)	169 (6.77)	158 (6.22)	
4	1117 (8.83)	449 (13.98)	246 (8.05)	237 (7.43)	185 (5.98)	
**Body mass index, n (%)**						<0.001
<25	3118 (31.59)	730 (26.56)	670 (30.48)	814 (31.95)	904 (37.06)	
25-30	3514 (33.10)	911 (31.01)	790 (32.97)	947 (35.90)	866 (32.39)	
>30	3816 (35.31)	1231 (42.44)	910 (36.56)	868 (32.15)	807 (30.55)	
**Age, n(%)**						<0.001
<40	3335 (35.30)	620 (21.70)	610 (28.49)	909 (38.72)	1196 (50.97)	
40-60	3475 (39.11)	880 (39.07)	869 (43.33)	911 (39.51)	815 (35.03)	
>60	3638 (25.59)	1372 (39.23)	891 (28.18)	809 (21.77)	566 (14.00)	
**Poverty Income Ratio, n (%)**						<0.001
<1.5	3510 (23.47)	928 (22.60)	748 (20.51)	857 (22.28)	977 (28.14)	
1.5-3	2642 (24.14)	768 (25.63)	569 (22.33)	651 (23.63)	654 (24.80)	
>3	4296 (52.39)	1176 (51.77)	1053 (57.16)	1121 (54.09)	946 (47.05)	
**Gender, n (%)**						<0.001
Male	5373 (51.54)	1142 (39.05)	1124 (46.35)	1466 (54.53)	1641 (65.17)	
Female	5075 (48.46)	1730 (60.95)	1246 (53.65)	1163 (45.47)	936 (34.83)	
**Race, n (%)**						<.001
Mexican American	1612 (7.15)	328 (4.78)	373 (6.77)	427 (7.52)	484 (9.40)	
Other Hispanic	797 (4.46)	193 (3.45)	169 (3.54)	217 (4.92)	218 (5.76)	
Non-Hispanic White	5333 (72.54)	1566 (74.65)	1237 (74.58)	1314 (72.59)	1216 (68.67)	
Non-Hispanic Black	1901 (10.13)	634 (12.94)	428 (9.96)	443 (9.01)	396 (8.69)	
Other Race – Including Multi-Racial	805 (5.72)	151 (4.18)	163 (5.15)	228 (5.96)	263 (7.49)	
**Marital status, n (%)**						<0.001
Married	5743 (58.46)	1584 (59.95)	1356 (61.74)	1506 (60.35)	1297 (52.17)	
Widowed	840 (5.52)	369 (9.53)	185 (5.26)	165 (4.59)	121 (2.83)	
Divorced	1082 (9.90)	327 (10.85)	251 (9.95)	271 (9.94)	233 (8.92)	
Separated	315 (2.09)	88 (2.43)	81 (2.32)	69 (1.55)	77 (2.12)	
Never married	1710 (16.73)	340 (11.52)	348 (14.43)	425 (16.30)	597 (24.22)	
Living with partner	758 (7.30)	164 (5.72)	149 (6.30)	193 (7.26)	252 (9.74)	
**Smoking status, n (%)**						<0.001
No	5573 (53.45)	1579 (53.95)	1316 (56.38)	1399 (54.65)	1279 (49.14)	
Yes	4875 (46.55)	1293 (46.05)	1054 (43.62)	1230 (45.35)	1298 (50.86)	
**Education, n (%)**						<0.001
Less than high school	2440 (15.85)	667 (16.03)	517 (13.51)	585 (14.81)	671 (18.80)	
High school or equivalent	2373 (22.61)	626 (21.11)	532 (22.20)	588 (22.94)	627 (24.09)	
College or above	5635 (61.54)	1579 (62.86)	1321 (64.28)	1456 (62.25)	1279 (57.11)	
**Drinking status, n (%)**						<0.001
No	2854 (22.75)	966 (28.73)	670 (23.08)	639 (20.65)	579 (18.83)	
Yes	7594 (77.25)	1906 (71.27)	1700 (76.92)	1990 (79.35)	1998 (81.17)	
**Physical Activity Equivalent, n(%)**						<0.001
Low physical activity;	3916 (34.50)	1263 (41.39)	875 (33.84)	904 (31.40)	874 (31.59)	
High physical activity	6532 (65.50)	1609 (58.61)	1495 (66.16)	1725 (68.60)	1703 (68.41)	
**Hypertension, n (%)**						<0.001
No	5781 (59.55)	1350 (50.01)	1259 (57.53)	1533 (62.59)	1639 (67.46)	
Yes	4667 (40.45)	1522 (49.99)	1111 (42.47)	1096 (37.41)	938 (32.54)	
**Diabetes, n (%)**						<0.001
No	8391 (85.03)	2191 (80.67)	1903 (84.68)	2164 (86.79)	2133 (87.78)	
Yes	2057 (14.97)	681 (19.33)	467 (15.32)	465 (13.21)	444 (12.22)	
**Hyperlipidemia, n(%)**						0.818
No	2560 (25.29)	725 (26.02)	572 (25.53)	637 (24.80)	626 (24.89)	
Yes	7888 (74.71)	2147 (73.98)	1798 (74.47)	1992 (75.20)	1951 (75.11)	
**All-cause mortality, n(%)**						<0.001
No	8846 (88.32)	2181 (79.99)	2047 (90.35)	2296 (90.62)	2322 (92.24)	
Yes	1602 (11.68)	691 (20.01)	323 (9.65)	333 (9.38)	255 (7.76)	
**Cardiovascular mortality, n(%)**						<0.001
No	9949 (96.53)	2644 (93.37)	2266 (97.25)	2527 (97.19)	2512 (98.28)	
Yes	499 (3.47)	228 (6.63)	104 (2.75)	102 (2.81)	65 (1.72)	

Weighted means were calculated for continuous variables, and categorical variables were expressed as n (%). The chi-square test or Fisher’s exact test was used for comparisons between groups, with *P* < 0.05 considered statistically significant. Q, quartile. CKM, Cardiovascular-kidney-metabolic syndrome. BMI, body mass index. PIR, poverty income ratio. PNI, prognostic nutritional index.

### 3.2 Associations between PNI and CKM staging

#### 3.2.1 Logistic regression analysis.

Multiple logistic regression analyses were performed to assess the association between PNI and CKM syndrome staging, with results detailed in Table 3. Analyses were conducted using three progressively adjusted models.

**Table 3:** Association between PNI and CKM staging across models.

Variables	Model1		Model2		Model3	
	OR (95%CI)	*P*	OR (95%CI)	*P*	OR (95%CI)	*P*
**CKM Stage0**						
PNI	1.03 (1.01 ~ 1.04)	0.003	0.97 (0.95 ~ 0.99)	0.046	0.98 (0.95 ~ 1.00)	0.100
PNI Q1	Reference		Reference		Reference	
PNI Q2	2.06 (1.59 ~ 2.66)	<0.001	1.72 (1.23 ~ 2.39)	0.002	1.86 (1.33 ~ 2.60)	<0.001
PNI Q3	1.98 (1.51 ~ 2.59)	<0.001	1.17 (0.83 ~ 1.67)	0.374	1.28 (0.88 ~ 1.87)	0.202
PNI Q4	2.13 (1.66 ~ 2.74)	<0.001	0.94 (0.66 ~ 1.33)	0.728	1.05 (0.73 ~ 1.52)	0.779
*P* for trend	1.06 (1.04 ~ 1.09)	<0.001	0.97 (0.94 ~ 1.01)	0.134	0.98 (0.95 ~ 1.02)	0.387
**CKM Stage1**						
PNI	1.01 (1.01 ~ 1.02)	0.013	0.99 (0.97 ~ 1.00)	0.060	1.00 (0.98 ~ 1.02)	0.988
PNI Q1	Reference		Reference		Reference	
PNI Q2	1.28 (1.05 ~ 1.56)	0.018	1.08 (0.88 ~ 1.33)	0.464	1.12 (0.88 ~ 1.42)	0.357
PNI Q3	1.41 (1.16 ~ 1.72)	<0.001	1.06 (0.86 ~ 1.29)	0.589	1.17 (0.93 ~ 1.48)	0.181
PNI Q4	1.29 (1.07 ~ 1.54)	0.007	0.86 (0.71 ~ 1.05)	0.135	1.01 (0.81 ~ 1.25)	0.955
*P* for trend	1.02 (1.01 ~ 1.04)	0.006	0.98 (0.96 ~ 1.00)	0.064	1.00 (0.98 ~ 1.02)	0.916
**CKM Stage2**						
PNI	1.00 (0.99 ~ 1.01)	0.385	1.01 (1.00 ~ 1.03)	0.059	1.01 (0.99 ~ 1.02)	0.280
PNI Q1	Reference		Reference		Reference	
PNI Q2	0.92 (0.80 ~ 1.05)	0.207	1.01 (0.88 ~ 1.17)	0.849	0.98 (0.85 ~ 1.13)	0.782
PNI Q3	0.88 (0.76 ~ 1.02)	0.090	1.05 (0.90 ~ 1.24)	0.525	0.99 (0.84 ~ 1.16)	0.891
PNI Q4	0.97 (0.85 ~ 1.12)	0.703	1.28 (1.10 ~ 1.49)	0.002	1.17 (1.00 ~ 1.37)	0.060
*P* for trend	1.00 (0.98 ~ 1.01)	0.709	1.03 (1.01 ~ 1.04)	0.002	1.02 (1.00 ~ 1.03)	0.055
**CKM Stage3**						
PNI	1.00 (0.98 ~ 1.01)	0.852	1.00 (0.99 ~ 1.02)	0.662	0.99 (0.97 ~ 1.02)	0.597
PNI Q1	Reference		Reference		Reference	
PNI Q2	0.94 (0.68 ~ 1.30)	0.714	0.99 (0.70 ~ 1.39)	0.952	0.87 (0.62 ~ 1.22)	0.427
PNI Q3	1.07 (0.80 ~ 1.43)	0.659	1.15 (0.84 ~ 1.56)	0.393	0.98 (0.71 ~ 1.35)	0.919
PNI Q4	0.98 (0.74 ~ 1.28)	0.859	1.10 (0.78 ~ 1.55)	0.605	0.91 (0.65 ~ 1.26)	0.567
*P* for trend	1.00 (0.97 ~ 1.03)	0.971	1.01 (0.98 ~ 1.05)	0.508	0.99 (0.96 ~ 1.03)	0.732
**CKM Stage4**						
PNI	0.95 (0.92 ~ 0.97)	<0.001	1.00 (0.98 ~ 1.01)	0.759	0.99 (0.98 ~ 1.01)	0.467
PNI Q1	Reference		Reference		Reference	
PNI Q2	0.54 (0.44 ~ 0.66)	<0.001	0.74 (0.59 ~ 0.93)	0.010	0.71 (0.57 ~ 0.89)	0.004
PNI Q3	0.49 (0.40 ~ 0.61)	<0.001	0.82 (0.66 ~ 1.03)	0.088	0.76 (0.61 ~ 0.95)	0.020
PNI Q4	0.39 (0.31 ~ 0.50)	<0.001	0.82 (0.63 ~ 1.05)	0.123	0.73 (0.57 ~ 0.95)	0.020
*P* for trend	0.90 (0.88 ~ 0.93)	<0.001	0.98 (0.95 ~ 1.01)	0.138	0.97 (0.94 ~ 0.99)	0.022
**Advanced CKM**						
PNI	0.97 (0.95 ~ 0.99)	<0.001	1.00 (0.99 ~ 1.01)	0.871	0.99 (0.98 ~ 1.01)	0.402
PNI Q1	Reference		Reference		Reference	
PNI Q2	0.64 (0.54 ~ 0.76)	<0.001	0.79 (0.65 ~ 0.97)	0.027	0.72 (0.59 ~ 0.88)	0.002
PNI Q3	0.65 (0.53 ~ 0.79)	<0.001	0.93 (0.76 ~ 1.15)	0.506	0.82 (0.65 ~ 1.02)	0.080
PNI Q4	0.54 (0.46 ~ 0.65)	<0.001	0.92 (0.73 ~ 1.16)	0.498	0.77 (0.61 ~ 0.97)	0.032
*P* for trend	0.94 (0.92 ~ 0.96)	<0.001	1.00 (0.97 ~ 1.02)	0.715	0.98 (0.95 ~ 1.00)	0.073

Weighted logistic regression was used to assess the association between PNI and CKM stages, with progressively increasing adjustments for potential confounders. Model 1 was unadjusted, Model 2 was adjusted for age, gender, race, BMI, education, PIR, marital status, smoking status, alcohol use, and physical activity, while Model 3 included additional adjustments for hypertension, diabetes, and hyperlipidemia. OR and 95% CI are presented, with *P* < 0.05 considered statistically significant. PNI, prognostic nutritional index; CKM, Cardiovascular-kidney-metabolic syndrome; BMI, body mass index; PIR, poverty income ratio; OR, odds ratio; CI, confidence interval.

The unadjusted Model 1 revealed significant associations between PNI and several CKM stages. Specifically, each unit increase in PNI was associated with higher odds of CKM stage 0 (OR = 1.03, 95% CI: 1.01–1.04, *P* = 0.003) and stage 1 (OR = 1.01, 95% CI: 1.01–1.02, *P* = 0.013). In contrast, higher PNI was associated with significantly lower odds of CKM stage 4 (OR = 0.95, 95% CI: 0.92–0.97, *P* < 0.001) and advanced CKM (OR = 0.97, 95% CI: 0.95–0.99, *P* < 0.001).

After adjustment for demographic and lifestyle factors in Model 2, the associations with the early stages (0 and 1) were substantially attenuated. This trend continued in the fully adjusted Model 3, where the linear association between PNI as a continuous variable and all CKM stages was no longer statistically significant. However, analysis of PNI quartiles revealed distinct and stage-specific patterns. For the early stages (0 and 1), the initial positive associations were not sustained after full adjustment. In stage 0, only the second quartile (Q2) showed significantly higher odds compared to the reference (Q1) (OR = 1.86, *P* < 0.001), with no significant trend across quartiles (*P*-trend = 0.387). For stage 1, no significant quartile comparisons or trends were observed.

No significant associations or consistent trends were identified between PNI quartiles and the intermediate stages (2 and 3). In advanced stages, a significant threshold effect was observed for CKM stage 4. Participants in the higher PNI quartiles (Q2, Q3, Q4) had significantly lower odds compared to those in Q1, with a significant inverse trend across quartiles (*P*-trend = 0.022). A similar protective pattern was evident for advanced CKM, where significantly lower odds were observed in Q2 (OR = 0.72, *P* = 0.002) and Q4 (OR = 0.77, *P* = 0.032), although the overall trend across quartiles was not statistically significant (*P*-trend = 0.073).

#### 3.2.2 Restricted cubic spline regression.

**Fig 2:**
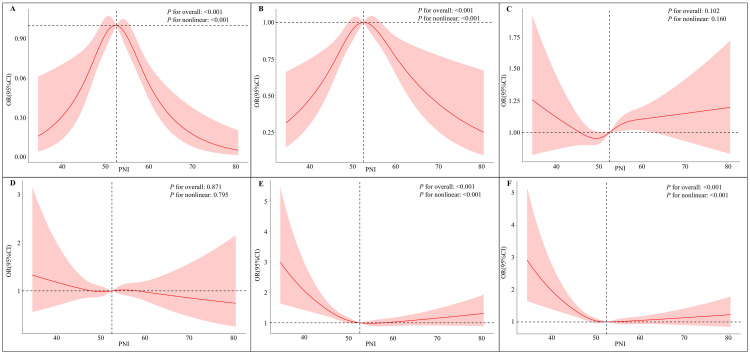
Non-linear Association Between PNI and CKM Staging. RCS regression was used to evaluate the non-linear relationship between PNI and CKM stages, adjusting for multiple potential confounders. The curve adjusts for age, gender, race, BMI, education, PIR, marital status, smoking status, alcohol use, physical activity, hypertension, diabetes, and hyperlipidemia. **(A)** CKM Stage 0. **(B)** CKM Stage 1. **(C)** CKM Stage 2. **(D)** CKM Stage 3. **(E)** CKM Stage 4. **(F)** Advanced CKM. RCS, restricted cubic spline; PNI, prognostic nutritional index; CKM, Cardiovascular-kidney-metabolic; OR, odds ratio; BMI, body mass index; PIR, poverty income ratio.

The non-linear relationships between PNI and each CKM stage, adjusted for all covariates in Model 3, were further visualized using RCS models (Fig 2A–2F).

Consistent with the logistic regression results, RCS curves revealed a significant inverted U-shaped relationship for the early stages. For CKM stage 0 (Fig 2A) and stage 1 (Fig 2B), the odds ratios were highest at moderate PNI levels (approximately 55–60), indicating the greatest risk within this range (*P*_non-linear < 0.001). Both lower and higher PNI values were associated with a reduced risk.

For the intermediate stages, the RCS analysis confirmed the absence of a significant association. The relationships between PNI and the odds of CKM stage 2 (Fig 2C) and stage 3 (Fig 2D) were not statistically significant (Overall *P* > 0.05).

In contrast, a significant and predominantly inverse relationship was observed for the advanced stages. For CKM stage 4 (Fig 2E) and advanced CKM (Fig 2F), the risk decreased sharply with increasing PNI at lower PNI values (*P*_non-linear < 0.001). The curves plateaued after a PNI of approximately 55–60, suggesting a ceiling effect for the protective benefit of PNI in severe disease.

### 3.3 Associations between PNI and all-cause/cardiovascular mortality

#### 3.3.1 Survival curve outcomes.

During a median follow-up of 123 months, a total of 10,448 individuals participated, including 1,602 all-cause mortality (11.68%) and 499 cardiovascular mortality (3.47%).

**Fig 3:**
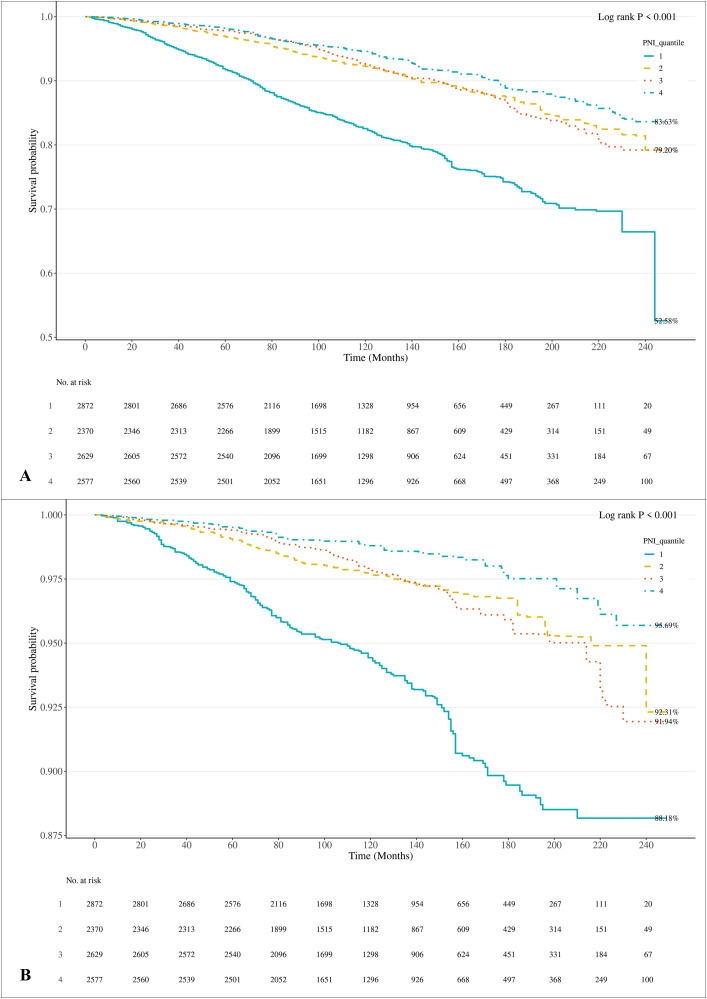
Kaplan – Meier Analysis of Cause-Specific Mortality in Advanced CKM. (A) All-cause mortality. **(B)** Cardiovascular mortality. Q1–Q4, PNI quartiles 1 - 4; PNI, prognostic nutritional index. CKM, Cardiovascular-kidney-metabolic syndrome.

For all-cause mortality (Fig 3A), significant differences in survival probabilities were observed among the four PNI quantiles. According to the log-rank test, the *P*-value (<0.001) indicates that the survival differences between the four groups are statistically significant. The survival probability of Q1 declined rapidly in the early follow-up period, which spanned approximately 200 months, but remained relatively stable and high in the subsequent period. By 240 months, the survival probability of Q1 had decreased to 52.58%, the lowest among the groups. The survival probabilities of Q2 and Q3 were relatively stable during the follow-up, with a survival probability of 79.2% at 240 months, slightly higher than Q1, with no significant difference between the two groups. The survival probability of Q4 remained stable and high, with a survival probability of 83.63% at 240 months, indicating that higher PNI quantiles were associated with a lower risk of all-cause mortality during the follow-up period.

For cardiovascular mortality (Fig 3B), significant differences in survival probabilities were also observed between the four groups (*P* < 0.001), and the results were similar to those for all-cause mortality. The survival probability of Q1 declined rapidly within the first 60 months, and in the later follow-up period (especially at 220 months), there was a significant decrease. At 240 months, the survival probability of Q1 was 88.18%, the lowest among all groups, indicating that this group had a higher risk of cardiovascular death. Q2 demonstrated a relatively stable survival probability during follow-up, with a survival probability of 92.31% at 240 months, indicating a lower cardiovascular mortality risk. The survival probability of Q3 was also stable, at 91.94% at 240 months, similar to Q2, indicating a low cardiovascular mortality risk but slightly higher than Q1. The survival probability of Q4 declined gradually during the follow-up period, reaching 95.69% at 240 months, significantly higher than the other groups. This suggests that higher PNI quantiles are associated with a reduced risk of cardiovascular mortality, especially in the later stages of follow-up.

#### 3.3.2 Multivariable hazard ratio analysis.

PNI exhibited a significant inverse relationship with all-cause mortality across all models (Model 1: HR = 0.92, *P* < 0.001; Model 2: HR = 0.97, *P* = 0.044; Model 3: HR = 0.97, *P* = 0.038). Compared to the reference quartile (PNI Q1), quartiles Q2, Q3, and Q4 consistently demonstrated significantly reduced risks in all models (all *P* < 0.001). Trend analysis for PNI quartiles showed significance in Model 1 (HR = 0.83, *P* < 0.001), but not in Models 2 and 3 (*P* = 0.731 and 0.811).

Similarly, PNI showed a significant inverse association with cardiovascular mortality across all models (Model 1: HR = 0.88, *P* < 0.001; Model 2: HR = 0.94, *P* < 0.001; Model 3: HR = 0.94, *P* < 0.001). Quartile comparisons revealed significantly lower mortality risk across Q2-Q4 relative to Q1, with all results significant (*P* ≤ 0.007). However, trend analysis indicated significance only in Model 1 (HR = 0.83, *P* < 0.001) but was nonsignificant in Models 2 and 3 (*P* = 0.802 and 0.634) (Table 4).

**Table 4:** Cox regression models for the association between PNI and mortality.

Variables	Model1		Model2		Model3	
	HR (95%CI)	*P*	HR (95%CI)	*P*	HR (95%CI)	*P*
**All-cause mortality**						
PNI	0.92 (0.90 - 0.94)	<0.001	0.97 (0.95 - 0.99)	0.044	0.97 (0.95 - 0.99)	0.038
PNI Q1	Reference		Reference		Reference	
PNI Q2	0.44 (0.37 - 0.53)	<0.001	0.60 (0.50 - 0.71)	<0.001	0.59 (0.50 - 0.70)	<0.001
PNI Q3	0.43 (0.37 - 0.51)	<0.001	0.70 (0.59 - 0.82)	<0.001	0.69 (0.59 - 0.81)	<0.001
PNI Q4	0.34 (0.29 - 0.40)	<0.001	0.73 (0.62 - 0.87)	<0.001	0.71 (0.59 - 0.85)	<0.001
*P* for trend	0.83 (0.79 - 0.86)	<0.001	1.02 (0.92 - 1.12)	0.731	1.01 (0.92 - 1.12)	0.811
**Cardiovascular mortality**						
PNI	0.88 (0.86 - 0.90)	<0.001	0.94 (0.92 - 0.96)	<0.001	0.94 (0.91 - 0.96)	<0.001
PNI Q1	Reference		Reference		Reference	
PNI Q2	0.38 (0.29 - 0.50)	<0.001	0.54 (0.40 - 0.71)	<0.001	0.52 (0.40 - 0.69)	<0.001
PNI Q3	0.39 (0.29 - 0.52)	<0.001	0.68 (0.52 - 0.90)	0.007	0.66 (0.50 - 0.88)	0.004
PNI Q4	0.22 (0.16 - 0.31)	<0.001	0.56 (0.41 - 0.75)	<0.001	0.53 (0.39 - 0.70)	<0.001
*P* for trend	0.83 (0.79 - 0.87)	<0.001	0.98 (0.84 - 1.14)	0.802	0.96 (0.82 - 1.13)	0.634

Model 1: Unadjusted. Model 2: Adjusted for age, gender, race, marital status, education, PIR, BMI, smoking status, drinking status, and physical activity equivalent. Model 3: Further adjusted for hypertension, diabetes, hyperlipidemia, alongside covariates in Model 2. HR: hazard ratio; CI: confidence interval; Q: quartile; PNI: prognostic nutritional index; CKM, Cardiovascular-kidney-metabolic syndrome; BMI, body mass index; PIR, poverty income ratio.

#### 3.3.3 Nonlinear associations.

**Fig 4:**
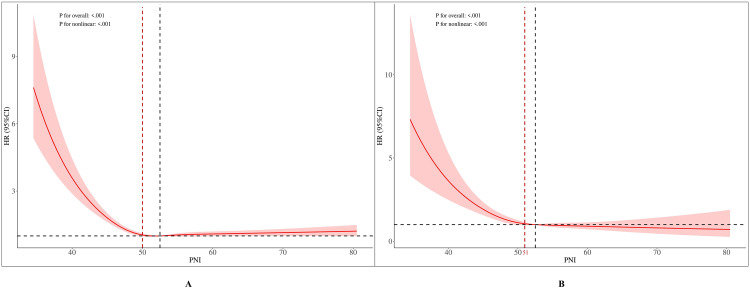
RCS Curves for the Association Between PNI and Mortality. The curves were adjusted for age, gender, race, marital status, education, PIR, BMI, smoking status, drinking status, physical activity equivalent, hypertension, diabetes, and hyperlipidemia. Vertical dashed lines indicate the threshold points derived from the two-piecewise linear regression model (PNI = 50 for all-cause mortality and PNI = 51 for cardiovascular mortality). The RCS curves show a similar but not identical inflection point, reflecting methodological differences. **(A)** All-cause mortality. **(B)** Cardiovascular mortality. RCS, restricted cubic spline; PNI: prognostic nutritional index; CKM, Cardiovascular-kidney-metabolic syndrome; HR, hazard ratio; BMI, body mass index; PIR, poverty income ratio.

Fig 4 presents the RCS curves depicting the relationship between PNI and mortality. Fig 4A illustrates the non-linear association between PNI and all-cause mortality. The curve is characterized by a sharp decline in the HR as PNI increases, with a clear inflection point observed around a PNI of 50. At PNI values below 50, the mortality risk decreases precipitously with each unit increase in PNI, indicating that individuals with low PNI levels are at a substantially elevated risk. The relationship is statistically significant (*P*_non-linear < 0.001), confirming PNI as a crucial predictor of all-cause mortality, where low PNI is a strong risk factor, and the protective effect of PNI plateaus at higher values.

Fig 4B displays a highly similar pattern for the relationship between PNI and cardiovascular mortality. The HR for cardiovascular mortality decreases sharply as PNI rises, particularly below a threshold of 50. Above this value, the risk reduction attenuates as the curve stabilizes, suggesting that the protective effect of PNI against cardiovascular mortality reaches a saturation point. The non-linear relationship is statistically significant (*P*_non-linear < 0.001), reinforcing that low PNI is a potent risk factor for cardiovascular death, while higher PNI levels are associated with sustained protective effects without evidence of harm.

#### 3.3.4 Threshold analysis.

A two-piecewise linear regression model was to analyze the relationship between the PNI and both all-cause and cardiovascular mortality.

For all-cause mortality, the standard linear regression model (Model 1) showed a significant inverse association between PNI and all-cause mortality (effect: 0.98, 95% CI: 0.97–0.99, *P* < 0.001), indicating that each unit increase in PNI is associated with a significant reduction in the risk of all-cause mortality. In contrast, the two-piecewise linear regression model (Model 2) demonstrated a clear threshold effect. Specifically, when PNI was below 50, an increase in PNI was significantly associated with a reduced risk of all-cause mortality (effect: 0.89, 95% CI: 0.87–0.92, *P* < 0.001). However, when PNI ≥ 50, the association was no longer statistically significant (effect: 1.00, 95% CI: 1.00–1.01, *P* = 0.108), suggesting that higher PNI levels beyond this threshold confer no additional survival benefit. The likelihood ratio test (*P* = 0.001) indicated that the two-piecewise model provided a significantly better fit than the standard linear model, further supporting the presence of a threshold effect.

**Table 5:** Threshold effect analysis of PNI on all-cause and cardiovascular mortality.

Outcome	Effect	*P*
**All-cause mortality**		
Model 1 Fitting model by standard linear regression	0.98 (0.97–0.99)	<0.001
Model 2 Fitting model by two-piecewise linear regression		
Inflection point	50	
<50	0.89 (0.87–0.92)	<0.001
≥50	1.00 (1.00–1.01)	0.108
*P* for likelihood test		<0.001
**Cardiovascular mortality**		
Model 1 Fitting model by standard linear regression	0.95 (0.93–0.97)	<0.001
Model 2 Fitting model by two-piecewise linear regression		
Inflection point	51	
<51	0.91 (0.87–0.94)	<0.001
≥51	0.99 (0.97–1.01)	0.440
*P* for likelihood test		0.001

The model was adjusted for age, gender, race, marital status, education, PIR, BMI, smoking status, drinking status, physical activity equivalent, hypertension, diabetes, and hyperlipidemia. PNI: prognostic nutritional index, CKM: Cardiovascular-kidney-metabolic syndrome; BMI, body mass index; PIR, poverty income ratio.

**Fig 5:**
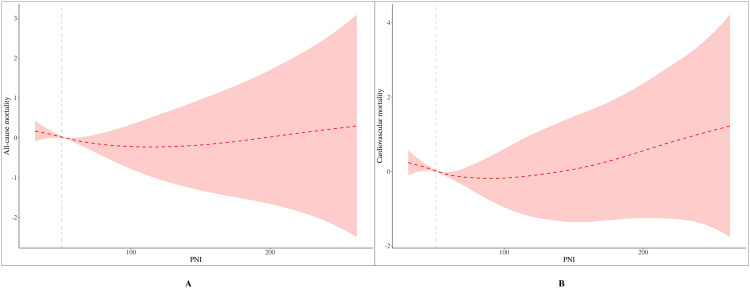
Threshold effect analysis of PNI on all-cause, and cardiovascular mortality. **(A)** All-cause mortality. **(B)** Cardiovascular mortality. PNI: prognostic nutritional index, CKM: Cardiovascular-kidney-metabolic syndrome.

A similar pattern was observed for cardiovascular mortality. The standard linear regression model showed a significant inverse association between PNI and cardiovascular mortality (effect: 0.95, 95% CI: 0.93–0.97, *P* < 0.001). In the two-piecewise linear regression analysis, when PNI was below 51, cardiovascular mortality risk decreased significantly with increasing PNI (effect: 0.91, 95% CI: 0.87–0.94, *P* < 0.001). However, when PNI ≥ 51, the risk plateaued, with no significant association observed (effect: 0.99, 95% CI: 0.97–1.01, *P* = 0.440), with no statistically significant change, indicating that higher PNI levels have little effect on cardiovascular mortality. The likelihood ratio test (*P* = 0.001) again confirmed the superiority of the two-piecewise model, supporting the threshold effect of PNI on cardiovascular mortality (Table 5 and Fig 5).

Both the two-piecewise linear regression and RCS analyses confirmed a threshold effect of PNI on mortality. The statistical model pinpointed the inflection points at PNI = 50 for all-cause mortality and PNI = 51 for cardiovascular death. Consistent with this, the RCS curves visually exhibited their steepest decline within this range. Together, these findings robustly indicate that PNI levels below approximately 50 are associated with a sharply increased risk of mortality.

#### 3.3.5 Subgroup analysis.

Tables 6 and 7 present the results of the subgroup analysis investigating the relationship between the PNI and mortality across various patient characteristics in individuals with CKM. The HR and 95% CI for different subgroups are provided, with statistical significance evaluated using the *P*-value. Additionally, the *P*-value for interaction is included to assess the heterogeneity of the effect of PNI on mortality across subgroups.

**Table 6:** Subgroup analysis of the association between PNI and all-cause mortality.

Variables	n (%)	HR (95%CI)	*P*	*P* for interaction
All patients	10448 (100.00)	0.88 (0.86 ~ 0.90)	<0.001	
**Gender, n (%)**				0.003
Male	5373 (51.43)	0.84 (0.82 ~ 0.87)	<0.001	
Female	5075 (48.57)	0.91 (0.88 ~ 0.95)	<0.001	
**Race, n (%)**				0.046
Mexican American	1612 (15.43)	0.90 (0.85 ~ 0.96)	0.001	
Other Hispanic	797 (7.63)	0.85 (0.76 ~ 0.94)	0.002	
Non-Hispanic White	5333 (51.04)	0.88 (0.86 ~ 0.91)	<0.001	
Non-Hispanic Black	1901 (18.19)	0.90 (0.85 ~ 0.95)	<0.001	
Other Race – Including Multi-Racial	805 (7.70)	0.76 (0.68 ~ 0.84)	<0.001	
**Marital status, n (%)**				0.057
Married	5743 (54.97)	0.89 (0.86 ~ 0.93)	<0.001	
Widowed	840 (8.04)	0.92 (0.88 ~ 0.97)	0.003	
Divorced	1082 (10.36)	0.96 (0.87 ~ 1.06)	0.430	
Separated	315 (3.01)	0.93 (0.86 ~ 1.01)	0.075	
Never married	1710 (16.37)	0.82 (0.77 ~ 0.86)	<0.001	
Living with partner	758 (7.25)	0.86 (0.78 ~ 0.96)	0.005	
**Smoking status, n (%)**				0.676
No	5573 (53.34)	0.87 (0.84 ~ 0.91)	<0.001	
Yes	4875 (46.66)	0.88 (0.85 ~ 0.91)	<0.001	
**Education, n (%)**				0.940
Less than high school	2440 (23.35)	0.88 (0.85 ~ 0.92)	<0.001	
High school or equivalent	2373 (22.71)	0.87 (0.84 ~ 0.91)	<0.001	
College or above	5635 (53.93)	0.88 (0.85 ~ 0.91)	<0.001	
**Drinking status, n (%)**				0.108
No	2854 (27.32)	0.91 (0.88 ~ 0.95)	<0.001	
Yes	7594 (72.68)	0.87 (0.84 ~ 0.90)	<0.001	
**Physical Activity Equivalent, n(%)**				0.581
Low physical activity;	3916 (37.48)	0.89 (0.87 ~ 0.92)	<0.001	
High physical activity	6532 (62.52)	0.88 (0.84 ~ 0.92)	<0.001	
**Hypertension, n (%)**				0.488
No	5781 (55.33)	0.88 (0.84 ~ 0.93)	<0.001	
Yes	4667 (44.67)	0.90 (0.87 ~ 0.92)	<0.001	
**Diabetes, n (%)**				0.976
No	8391 (80.31)	0.89 (0.86 ~ 0.91)	<0.001	
Yes	2057 (19.69)	0.89 (0.85 ~ 0.92)	<0.001	
**Hyperlipidemia, n(%)**				0.260
No	2560 (24.50)	0.86 (0.81 ~ 0.90)	<0.001	
Yes	7888 (75.50)	0.88 (0.86 ~ 0.91)	<0.001	
**Body mass index, n (%)**				0.010
<25	3118 (29.84)	0.84 (0.81 ~ 0.88)	<0.001	
25-30	3514 (33.63)	0.93 (0.89 ~ 0.97)	<0.001	
>30	3816 (36.52)	0.88 (0.84 ~ 0.91)	<0.001	
**Age, n(%)**				0.238
<40	3335 (31.92)	1.03 (0.91 ~ 1.16)	0.662	
40-60	3475 (33.26)	0.89 (0.82 ~ 0.97)	0.009	
>60	3638 (34.82)	0.92 (0.89 ~ 0.95)	<0.001	
**Poverty Income Ratio, n (%)**				0.855
<1.5	3510 (33.59)	0.88 (0.85 ~ 0.92)	<0.001	
1.5-3	2642 (25.29)	0.87 (0.84 ~ 0.91)	<0.001	
>3	4296 (41.12)	0.88 (0.85 ~ 0.92)	<0.001	

HR: hazard ratio, CI: confidence interval, PNI: prognostic nutritional index, CKM: Cardiovascular-kidney-metabolic syndrome.

**Table 7:** Subgroup analysis of the association between PNI and cardiovascular mortality.

Variables	n (%)	HR (95%CI)	*P*	*P* for interaction
All patients	10448 (100.00)	0.88 (0.86 ~ 0.90)	<0.001	
**Gender, n (%)**				0.003
Male	5373 (51.43)	0.84 (0.82 ~ 0.87)	<0.001	
Female	5075 (48.57)	0.91 (0.88 ~ 0.95)	<0.001	
**Race, n (%)**				0.046
Mexican American	1612 (15.43)	0.90 (0.85 ~ 0.96)	0.001	
Other Hispanic	797 (7.63)	0.85 (0.76 ~ 0.94)	0.002	
Non-Hispanic White	5333 (51.04)	0.88 (0.86 ~ 0.91)	<0.001	
Non-Hispanic Black	1901 (18.19)	0.90 (0.85 ~ 0.95)	<0.001	
Other Race – Including Multi-Racial	805 (7.70)	0.76 (0.68 ~ 0.84)	<0.001	
**Marital status, n (%)**				0.057
Married	5743 (54.97)	0.89 (0.86 ~ 0.93)	<0.001	
Widowed	840 (8.04)	0.92 (0.88 ~ 0.97)	0.003	
Divorced	1082 (10.36)	0.96 (0.87 ~ 1.06)	0.430	
Separated	315 (3.01)	0.93 (0.86 ~ 1.01)	0.075	
Never married	1710 (16.37)	0.82 (0.77 ~ 0.86)	<0.001	
Living with partner	758 (7.25)	0.86 (0.78 ~ 0.96)	0.005	
**Smoking status, n (%)**				0.676
No	5573 (53.34)	0.87 (0.84 ~ 0.91)	<0.001	
Yes	4875 (46.66)	0.88 (0.85 ~ 0.91)	<0.001	
**Education, n (%)**				0.940
Less than high school	2440 (23.35)	0.88 (0.85 ~ 0.92)	<0.001	
High school or equivalent	2373 (22.71)	0.87 (0.84 ~ 0.91)	<0.001	
College or above	5635 (53.93)	0.88 (0.85 ~ 0.91)	<0.001	
**Drinking status, n (%)**				0.108
No	2854 (27.32)	0.91 (0.88 ~ 0.95)	<0.001	
Yes	7594 (72.68)	0.87 (0.84 ~ 0.90)	<0.001	
**Physical Activity Equivalent, n(%)**				0.581
Low physical activity;	3916 (37.48)	0.89 (0.87 ~ 0.92)	<0.001	
High physical activity	6532 (62.52)	0.88 (0.84 ~ 0.92)	<0.001	
**Hypertension, n (%)**				0.488
No	5781 (55.33)	0.88 (0.84 ~ 0.93)	<0.001	
Yes	4667 (44.67)	0.90 (0.87 ~ 0.92)	<0.001	
**Diabetes, n (%)**				0.976
No	8391 (80.31)	0.89 (0.86 ~ 0.91)	<0.001	
Yes	2057 (19.69)	0.89 (0.85 ~ 0.92)	<0.001	
**Hyperlipidemia, n(%)**				0.260
No	2560 (24.50)	0.86 (0.81 ~ 0.90)	<0.001	
Yes	7888 (75.50)	0.88 (0.86 ~ 0.91)	<0.001	
**Body mass index, n (%)**				0.010
<25	3118 (29.84)	0.84 (0.81 ~ 0.88)	<0.001	
25-30	3514 (33.63)	0.93 (0.89 ~ 0.97)	<0.001	
>30	3816 (36.52)	0.88 (0.84 ~ 0.91)	<0.001	
**Age, n(%)**				0.238
<40	3335 (31.92)	1.03 (0.91 ~ 1.16)	0.662	
40-60	3475 (33.26)	0.89 (0.82 ~ 0.97)	0.009	
>60	3638 (34.82)	0.92 (0.89 ~ 0.95)	<0.001	
**Poverty Income Ratio, n (%)**				0.855
<1.5	3510 (33.59)	0.88 (0.85 ~ 0.92)	<0.001	
1.5-3	2642 (25.29)	0.87 (0.84 ~ 0.91)	<0.001	
>3	4296 (41.12)	0.88 (0.85 ~ 0.92)	<0.001	

HR: hazard ratio, CI: confidence interval, PNI: prognostic nutritional index, CKM: Cardiovascular-kidney-metabolic syndrome.

The subgroup analysis reveals that the relationship between PNI and mortality, including cardiovascular mortality, varies across gender, race, and BMI. Significant interactions were observed between gender and BMI with the effect of PNI on mortality, with stronger associations found in males and individuals with lower BMI. These findings underscore the importance of considering individual patient characteristics when using PNI as a prognostic marker for mortality in clinical practice.

## 4 Discussion

This study, encompassing 10,448 participants, is the first to establish the association between PNI and different stages of CKM syndrome, as well as its predictive value for mortality in the general CKM population. Key findings indicate that PNI exhibits a stage-specific association with CKM, demonstrates a nonlinear relationship with all-cause and cardiovascular mortality, and serves as an independent predictor of mortality risk with an identified threshold around 50.

### 4.1 Stage-specific associations between PNI and CKM syndrome

Our analysis revealed a complex relationship between PNI and CKM stages. A notable inverted U-shaped association was observed in early stages (0–1), while no significant association was found for stage 2 and 3. Conversely, a strong inverse relationship was evident in CKM stage4 and advanced CKM, with higher PNI conferring a clear protective effect.

The inverted U-shaped relationship in early CKM suggests that moderate PNI levels may paradoxically reflect the highest risk. We hypothesize this could represent a subclinical state of ineffective metabolic-immune activation, whereby the PNI extremes might indicate more stable physiological conditions, specifically low PNI corresponding to classical malnutrition and high PNI to preserved regulatory capacity [29]. This novel finding requires cautious interpretation and validation in future studies to rule out confounding.

No significant association was observed in stages 2–3. This may be attributed to the progression of the disease at these stages, where the core pathophysiology shifts towards overt organ damage and metabolic dysregulation. Nutritional and immune statuses are influenced by a complex interplay of factors beyond PNI alone, which may not fully capture the extent of metabolic disturbances and organ injury, thus limiting its prognostic accuracy during this phase.

In contrast, for CKM Stage 4 and advanced CKM, where multi-organ dysfunction and a systemic inflammatory state prevail [30,31], our findings demonstrate a clear inverse association with PNI. This protective effect suggests that in advanced disease, robust nutritional status and preserved immune competence are critical for mitigating pathological progression and sustaining organ function. We hypothesize that adequate nutritional reserves help counteract the catabolic state and support tissue repair, while a sufficient lymphocyte count indicates retained immune regulatory capacity, potentially tempering the excessive inflammation that drives CKM progression.

### 4.2 PNI as a predictor of mortality in CKM syndrome

A pivotal finding is that PNI is a robust, independent predictor of both all-cause and cardiovascular mortality, with identified risk thresholds at PNI ≈ 50 and ≈51, respectively. This aligns with established evidence that malnutrition accelerates the progression of cardiovascular and renal diseases [32,33]. Low PNI, reflecting both malnutrition and immune dysfunction, may lead to reduced disease resistance, exacerbated myocardial dysfunction, and heightened inflammatory responses, collectively increasing mortality risk [33–37]. It is critical to emphasize that the observational nature of this association cannot establish causality. While PNI is a powerful prognostic marker, it remains unclear whether improving PNI through nutritional interventions would directly improve CKM outcomes. Further refining its prognostic utility, we found the association between PNI and mortality to be nonlinear, highlighting the dual burden of undernutrition and overnutrition [38–43]. This finding positions PNI alongside other nutritional-inflammatory indices relevant to cardiometabolic and renal health, such as the controlling nutritional status (CONUT) score, low albumin-to-neutrophil ratio (ANLR), systemic inflammation response index (SIRI), systemic immune-inflammation index (SII) and nutritional risk index (NRI), and underscores the need for a multi-marker approach to risk stratification [44–49].

### 4.3 Subgroup variations

Subgroup analyses revealed that the protective association of PNI was more pronounced in males and individuals with a BMI < 25. Variations were also observed across racial groups. These interactions suggest that the prognostic power of PNI is modified by physiological and social factors, a notion supported by existing literature on demographic disparities in CKM [50–52], underscoring the necessity for personalized assessment.

### 4.4 Strengths and limitations

The primary strength of this study lies in its novel investigation into the stage-specific and prognostic role of PNI in CKM syndrome, utilizing a large, nationally representative cohort from NHANES. This is the first study to delineate the complex, stage-dependent associations and to identify clear thresholds for mortality risk, providing a valuable foundation for the use of PNI as a simple, cost-effective risk stratification tool in the CKM population.

However, several limitations must be considered when interpreting these findings. The cross-sectional design of the staging analysis and the observational nature of the mortality follow-up preclude any causal inferences between PNI and CKM outcomes. Furthermore, the exclusion of a substantial number of initial participants due to missing data, while common in NHANES analyses, introduces the potential for selection bias if the excluded individuals differed systematically from the analytical sample. The PNI, as a composite of only serum albumin and lymphocyte count, offers clinical practicality but may not fully capture the complexity of nutritional status and chronic inflammation, especially when compared to other established indices. The generalizability of our findings to non-U.S. populations may be limited, and the intriguing non-linear relationships and specific PNI thresholds identified require validation in dedicated prospective studies.

## 5 Conclusion

PNI is closely linked to both the disease staging of CKM syndrome and patient mortality risk, and serves as an independent predictor of all-cause and cardiovascular death in CKM patients, with a clear risk threshold identified. As a simple and cost-effective indicator, PNI can be used in clinical practice to identify high-risk CKM patients. However, due to the observational nature of this study, it demonstrates an association rather than causation. Future interventional studies are needed to confirm whether improving nutritional and immune status can directly enhance clinical outcomes for CKM patients.

## Supporting information

S1 Table Defining criteria for CKM stages using NHANES variables. This table details the specific variable definitions, operational thresholds, and criteria adapted from AHA framework to define each CKM syndrome stage (0–4) within the NHANES dataset. It includes the corresponding NHANES variable codes and the implementation logic for risk factors such as adiposity, dysglycemia, blood pressure, CKD, and CVD. The supplementary methods below the table provide specifics on the calculation of estimated glomerular filtration rate (eGFR), urinary albumin-to-creatinine ratio (UACR), 10-year CVD risk, metabolic syndrome (MetS), and clinical CVD identification.
(DOCX)

## Abbreviations

CKM: Cardiovascular-kidney-metabolic
PNI: Prognostic nutritional index
NHANES: National Health and Nutrition Examination Survey
RCS: Restricted cubic spline
AHA: American Heart Association
CKD: Chronic kidney disease
CVD: Cardiovascular diseases
NDI: National Death Index
NCHS: National Center for Health Statistics
PIR: Poverty-income ratio
BMI: Body mass index
SBP: Systolic blood pressure
DBP: Diastolic blood pressure
TC: Total cholesterol
TG: Triglycerides
LDL-C: Low-density lipoprotein cholesterol
HDL-C: High-density lipoprotein cholesterol
ANOVA: Analysis of variance
SIRI: Systemic inflammatory response index
SII: Systemic immune-inflammation index
NRI: Nutritional risk index
ANLR: Low albumin-to-neutrophil ratio
Q: Quartile
OR: Odds ratios
CI: Confidence interval
HR: Hazard Ratio.
