# Supplementary material for: Prognostic nutritional index predicts staging and mortality in cardiovascular-kidney-metabolic syndrome
Source: PLoS One. 2025 Dec 18;20(12):e0338084. doi: 10.1371/journal.pone.0338084 (PMC12714200; doi:10.1371/journal.pone.0338084)
Supplement: S1 Table — This table details the specific variable definitions, operational thresholds, and criteria adapted from AHA framework to define each CKM syndrome stage (0–4) within the NHANES dataset. It includes the corresponding NHANES variable codes and the implementation logic for risk factors such as adiposity, dysglycemia, blood pressure, CKD, and CVD. The supplementary methods below the table provide specifics on the calculation of estimated glomerular filtration rate (eGFR), urinary albumin-to-creatinine ratio (UACR), 10-year CVD risk, metabolic syndrome (MetS), and clinical CVD identification. (DOCX) [file pone.0338084.s001.docx]

**Supplementary Table 1: Defining Criteria for CKM Stages Using NHANES Variables**

| **CKM Stage** | **Definition (AHA)** | **NHANES Variables Used** | **Threshold / Criteria Implemented in This Study** |
| --- | --- | --- | --- |
| **Stage 0** | No CKM risk factors | BMI: BMXBMI Waist circ.: BMXWAIST Glucose: LBXGLU HbA1c: LBXGH BP: BPXSY1, BPXDI1 CKD: eGFR, URXUMA, URXUCR CVD: MCQ160b–f | BMI < 25 kg/m² Waist < 88 cm (F) or < 102 cm (M) Fasting glucose < 100 mg/dL or HbA1c < 5.7% SBP < 130 mmHg and DBP < 80 mmHg No self-reported diabetes or BP meds eGFR ≥ 60 ml/min/1.73m² and UACR < 30 mg/g No CVD history (all MCQ160b–f = 2) |
| **Stage 1** | Presence of overweight/obesity or dysfunctional adiposity, without other metabolic risk factors or CKD. | BMI: BMXBMI Waist circ.: BMXWAIST Glucose: LBXGLU HbA1c: LBXGH BP: BPXSY1, BPXDI1 CKD: eGFR, URXUMA, URXUCR CVD: MCQ160b–f | BMI ≥ 25 kg/m² OR waist ≥ 88 cm (F) / 102 cm (M) OR prediabetes: glucose 100–124 mg/dL OR HbA1c 5.7–6.4% AND no diagnosed diabetes (DIQ010=2 and no insulin/oral hypoglycaemic use) AND not meeting Stage 2–4 criteria |
| **Stage 2** | Presence of metabolic risk factors or CKD. | TG: LBXSTR BP: BPXSY1, BPXDI1, MCQ080a DM: DIQ050, DIQ010, LBXGLU, LBXGH MetS: derived from above CKD: eGFR,UACR | TG ≥ 135 mg/dL  BP ≥ 130/80 mmHg OR on BP meds  Diabetes: glucose ≥ 125 mg/dL OR HbA1c ≥ 6.5% OR self-report DM OR on insulin/oral meds  MetS: ≥3 ATP-III criteria  CKD: KDIGO moderate/high risk (eGFR 30–59 OR UACR 30–299 mg/g)  AND not Stage 3–4 |
| **Stage 3** | Subclinical CVD or very-high-risk CKD OR high 10-y CVD risk  Presence of subclinical CVD | CKD: eGFR, UACR 10-y risk: age, sex, SBP, DBP, TC, HDL, DM, smoking, BP-meds, eGFR Biomarkers: LBXHCTN, LBXNTProBNP | Subclinical CVD or very-high-risk CKD OR high 10-year CVD risk Presence of subclinical CVD Very-high-risk CKD: eGFR < 30 ml/min/1.73 m² or UACR ≥ 300 mg/g High-sensitivity cTn or NT-proBNP above the 95th percentile for each sex (i.e., sex-specific 95th percentile) |
| **Stage 4** | Presence of clinical CVD | MCQ160b–f | Self-reported diagnosis of: Coronary heart disease Angina Heart attack Heart failure Stroke ± kidney failure (eGFR <15 mL/min/1.73 m²; dialysis data unavailable in NHANES, so only eGFR <15 was used as a proxy). |

**Supplementary Methods：**

eGFR was calculated with the 2021 CKD-EPI creatinine equation (race-free) using serum creatinine (LBXSCRSAU).

UACR was computed as URXUMA / URXUCR (mg/g).

1. year CVD risk ≥ 20 % was estimated with a Cox model incorporating age, sex, SBP, DBP, total cholesterol, HDL-C, diabetes status, current smoking, eGFR and antihypertensive-medication use (internal derivation, NHANES 1999–2018).

MetS was defined by ATP-III criteria: waist ≥88 cm (F) /102 cm (M), TG ≥150 mg/dL, HDL-C <50 mg/dL (F) /<40 mg/dL (M), BP ≥ 130/80 mmHg or on antihypertensive medication, fasting glucose ≥100 mg/dL; ≥3 components positive.

Clinical CVD was identified by an affirmative answer to any of MCQ160b–f.
